# Intrinsic capacity, functional and psychosocial aspects of older adults participating in a multicomponent physical exercise program

**DOI:** 10.3389/fragi.2025.1562383

**Published:** 2025-06-23

**Authors:** Sarah Giulia Bandeira Felipe, Clarisse Bielh Printes, Fabiane de Oliveira Brauner, Douglas Katusohi Sato, Rafael Reimann Baptista

**Affiliations:** ^1^ Graduate Program in Biomedical Gerontology, Pontifical Catholic University of Rio Grande do Sul (PUCRS), Porto Alegre, Brazil; ^2^ Institute of Geriatrics and Gerontology, Pontifical Catholic University of Rio Grande do Sul (PUCRS), Porto Alegre, Brazil; ^3^ BioHub Innovation Laboratory, Pontifical Catholic University of Rio Grande do Sul (PUCRS), Porto Alegre, Brazil

**Keywords:** intrinsic capacity, older adults, physical exercise, healthy aging, quality of life

## Abstract

**Introduction:**

In 2015, the WHO introduced intrinsic capacity (IC) as a health indicator with five domains to promote healthy aging. Multicomponent exercise programs are recommended to enhance IC, but research in Brazil on their comprehensive impact is limited. This study aimed to evaluate the effects of such a program on IC, functional, and psychosocial aspects in older adults.

**Methods:**

This pre- and post-study assessed older adults in Brazil enrolled in a multicomponent training program, evaluating IC as the main outcome using specific tests for each domain. Inclusion criteria were: aged 60+, completing assessments in five domains, attending the program at least twice a week, and participating in two exercise modalities per session for 90 min. Exclusion criteria included: history of stroke, Parkinson’s or Alzheimer’s, recent hand, hip, or knee surgery, or absence for more than 15 consecutive days. A total of 43 older adults were evaluated, and the score was calculated by summing the results of the five domains, yielding a total score ranging from 0 to 10 points. Subsequently, participants underwent a 12-week intervention involving multicomponent exercises and were reassessed.

**Results:**

After 12 weeks of intervention, there was a significant reduction in the proportion of participants with low IC, from 7.0% to 0.0%, and an increase in those with high IC, from 4.7% to 20.0% (p = 0.018). Improvements were seen in cognitive aspects, locomotor dimension (p < 0.001), vitality (p = 0.045) and functional classification (p < 0.001), with the greatest effect in the locomotor domain (es = 1.12). Significant gains were also observed in perceived health, quality of life, and physical activity (p < 0.002; p < 0.004; p < 0.001). Body composition showed improvements, including reduced body fat percentage, increased muscle mass, and better fat classification (p < 0.001), along with reductions in waist and abdominal circumferences (p < 0.001; p = 0.001).

**Conclusion:**

The multicomponent exercise program demonstrated a positive influence on composite IC, including functional and psychosocial aspects. These findings highlight the critical role of tailored and supervised exercise interventions in enhancing both physical and psychosocial dimensions of health, contributing to healthier aging trajectories.

## 1 Introduction

Intrinsic capacity (IC), a concept introduced by the World Health Organization (WHO) in 2015, represents the composite of an individual’s physical and mental capacities. As a multidimensional health indicator, IC is integral to the WHO’s framework for healthy aging and is categorized into five domains: cognitive, psychological, sensory, locomotor, and vitality (World Health Organization, 2020).

This paradigm shifts the focus from traditional geriatrics, which primarily addresses diseases and deficits, to a function-centered approach that emphasizes the abilities of older adults irrespective of their age or health conditions (Hu et al., 2023; Yu et al., 2021). IC encompasses the dynamic interaction between physical and mental capacities and environmental factors, influencing how individuals perceive and experien. A study conducted with 941 Brazilian elderly individuals, followed over a period of 58 months, revealed an average score of 5.8 points in intrinsic capacity. During the follow-up period, it was observed that for each increase in the intrinsic capacity score, there was a 21% reduction in the risk of mortality (Cabral et al., 2024). Regarding functional and psychosocial aspects, a lower intrinsic capacity score is associated with a 7% increased risk of dependence for activities of daily living (ADL). Moreover, low quality of life contributes to a decline in intrinsic capacity by one point and increases the risk of long-term care admissions by 6% (Zhou et al., 2023).

Thus, assessing the various domains of intrinsic capacity, whether separately or together, is important for predicting future declines that an older person may experience, as well as mortality, in comparison to simply measuring diseases or morbidities. Furthermore, it provides an opportunity to identify interventions aimed at promoting healthy aging that can address the needs, preferences, and goals of each older individual in their environment (World Health Organization, 2020).

In this context, multicomponent exercises, combining aerobic, resistance, flexibility, and balance training, emerge as a promising strategy. This intervention is recommended by the Integrated Care for Older People (ICOPE) Manual to maintain and optimize various domains (World Health Organization, 2019).

Evidence further suggests that these programs help maintain mobility, musculoskeletal function, and the optimal functioning of other body systems (neurological, cardiovascular, respiratory, digestive, endocrine), all of which impact intrinsic capacity. However, the effects of multicomponent exercise may vary based on baseline fitness levels, exercise adherence, or comorbidities, highlighting the importance of personalizing interventions to meet individual needs and maximize their benefits (Izquierdo, 2018).

Additionally, participating in group-based exercise fosters social connections, reinforcing psychosocial wellbeing and societal integration (Yu et al., 2024). These practices contribute to physical autonomy and improve quality of life, key determinants of aging well (Pinheiro et al., 2022).

The Decade of Healthy Aging emphasizes the urgency of implementing measures to improve the capacities of older adults. Despite this, research on the comprehensive impact of multicomponent exercise remains scarce in Brazil, and to date, no study has assessed the effect of this intervention in the country. Addressing this gap, the present study aims to primarily evaluate changes in intrinsic capacity and, secondarily, assess functional and psychosocial outcomes in older adults participating in a multicomponent physical exercise program. Key research questions include: How to develop a valid composite IC score? Which IC domains are most responsive to the multicomponent training program? Does the exercise program significantly modify the composite IC score? Is there an association between IC changes and functional/psychosocial outcomes?

The hypothesis of this study is that a 12-week multicomponent physical exercise program has effects on improving intrinsic capacity, including functional and psychosocial aspects, among older adults.

It is expected that the results of this research will provide a solid foundation for the development and implementation of health policies and programs aimed at promoting healthy aging. By optimizing existing resources and focusing on their reorganization, these strategies can reduce the need for high investments while enhancing the capacities of older adults in their environment. In line with the ICOPE model (World Health Organization, 2019), the study highlights the potential of reorienting and integrating available health services to more effectively meet the needs of this age group. Furthermore, it aligns with the Decade of Healthy Aging, particularly in Priority Action Area II, which aims to ensure that communities promote the capacities of older adults. At the national level, the program also reinforces the guidelines of the National Policy for the Elderly, focusing on the prevention, promotion, protection, and recovery of health, contributing to the reduction of diseases, improved functionality and quality of life, and fostering greater longevity.

## 2 Methods

This study was approved by the Research Ethics Committee of the Pontifical Catholic University of Rio Grande do Sul (PUCRS) under approval number 5.517.315/CAAE: 60234322.1.0000.5336. The ethical guidelines proposed by the Helsinki Declaration and the norms set forth in Resolution 466/12 of the National Health Council for research involving human beings were followed, with the respective signing of the Informed Consent Term (ICF).

This is a pre- and post-study with mixed interventions involving apparently healthy elderly individuals enrolled in a Multicomponent Physical Exercise Program in Porto Alegre, Brazil. This study design was chosen because all the elderly participants in the program were already engaging in physical exercise, making randomization impossible. If participant allocation had been used, the control group elderly individuals could, eventually, have contact with those in the intervention group, which would contaminate the results of this research. The TREND checklist from the EQUATOR Network was employed to guide the methodology. The protocol for conducting the study is available at: https://doi.org/10.53886/gga.e0000104_EN.

It is noteworthy that this study did not involve the random allocation of participants into intervention and control groups. Instead, participants autonomously selected the modalities, frequency, and duration of the activities provided by the program. Although these modalities adhered to standardized implementation protocols, practical heterogeneity was inevitable due to variations in instructors and participant group composition. This methodological choice was intended to reflect the actual engagement patterns of older adults in community-based interventions, acknowledging that a fully controlled experimental design may not accurately represent real-world conditions. Accordingly, the study design aligns with the principles of a pragmatic clinical trial, which better accommodates the complexity and variability inherent to real-life healthcare settings, particularly in contexts where traditional randomized controlled trials are difficult to operationalize.

A non-probabilistic convenience sample was used, calculated based on a previous study by Sánchez-Sánchez, resulting in a total of 31 older adults, accounting for potential dropouts. Recruitment occurred through a Physical Exercise Program in community located in Porto Alegre, Brazil, *via* phone calls and messages, social media announcements, and the local team. A total of 112 individuals were invited between July and September 2023 to participate in the program, and of these, 48 accepted the invitation. The reasons for non-participation included refusal of the invitation, lack of response to the invitation, and other factors such as logistical issues and physical limitations.

Inclusion criteria were as follows: being aged 60 years or older; completing assessments across five domains (cognition, vitality, psychological, locomotor, and sensory); attending the program at least twice per week (24 sessions over 12 weeks); and participating in two exercise modalities per session for 90 min per day. Strength training and cardiovascular training were recommended for at least two sessions per week, while other activities could be performed up to three times per week.

The exclusion criteria were defined based on a thorough medical assessment and previous clinical reports to identify physical or cognitive impairments that could compromise or limit participation in the evaluations. The exclusion criteria included: a history of stroke, Parkinson’s disease, or Alzheimer’s disease, surgeries involving the hand, hip, or knee in the past 12 months, as well as absence from the program for more than 15 consecutive days. The specified period is justified by the importance of continuity and regularity of interventions to ensure adherence to the program. Prolonged absences could compromise the gains related to intrinsic capacity, hindering the participants’ progress and the maintenance of the benefits achieved. Furthermore, absences exceeding this interval may result in a loss of motivation and make it more difficult for participants to reintegrate into the program.

### 2.1 Procedures

Participants underwent two individual evaluation sessions: one at the start of the study (pre-intervention) and another after completing the 12-week multicomponent training program, conducted between September and December 2023. The following instruments were used for data collection: a questionnaire on sociodemographic, clinical, and social characteristics; the Geriatric Depression Scale (GDS-15) (Yesavage et al., 1982); the International Physical Activity Questionnaire (IPAQ) (Marshall and Bauman, 2001; Craig et al., 2003); the Short Form 6 Dimensions (SF-6D Brazil) for generic quality of life assessment (Brazier et al., 1998); the Functional Independence Measure (Rikli and Jones, 1991); and the Montreal Cognitive Assessment (MoCA) (Nasreddine et al., 2005; Freitas et al., 2013). Self-reported assessments of the sensory domain were conducted based on specific questions about vision and hearing, extracted from the ICOPE protocol. The questions included: “Do you wear glasses?” and “Do you have difficulty seeing far or near?” for vision, and “Do you use a hearing aid?” and “Do you have difficulty hearing whispers?” for hearing (World Health Organization, 2020). Intrinsic Capacity Domains were evaluated according to Table 1.

**TABLE 1:** Evaluation of intrinsic capacity domains.

Capacidade intrínseca	Categories	Instruments	Classification	Score
Vitality	Handgrip strength	Dynamometer	<27 kgf for men/< 16 kgf for women (Cruz-Jentoft et al., 2019) ≥27 kgf for men/< 16 kgf for women (Cruz-Jentoft et al., 2019)	0.0 0.5
	Body Mass Index (BMI)	Scale	<22 kg/m^2^ and > 27 kg/m^2^ (Lipschitz, 1994) ≥22 and ≤27 kg/m^2^ (Lipschitz et al., 1994)	0.0 0.5
	Weight loss	Have you had unintentional weight loss of 3 kg or more in the last 3 months? How many kg?	≥3 kg <3 kg	0.0 0.5
	Appetite loss	Have you experienced loss of appetite?	Yes No	0.0 0.5
Sensory	Vision	- Do you wear glasses? Do you have difficulty seeing far or near?	Reports difficulty seeing far or near and uses prescription glasses Does not have difficulty seeing far or near and does not use prescription glasses	0.0 1.0
	Hearing	- Do you use hearing aids? Do you have difficulty hearing whispers?	Reports difficulty hearing whispers and uses hearing aids Does not have difficulty hearing whispers and does not use hearing aids	0.0 1.0
Locomotion	Functional mobility	*Timed Up and Go Test*	>10 s (Podsiadlo and Richardson, 1991) <10 s (Podsiadlo and Richardson, 1991)	0.0 0.5
	Lower limb strength	Sit-to-Stand Test	Number of repetitions below the age- and sex-specific threshold Riklie and Jones, 1991; 2013) Number of repetitions equal to or above the age- and sex-specific threshold Riklie and Jones, 1991; 2013)	0.0 0.5
	Flexibility	Sit-and-Reach Test	Flexibility outside the recommended range for age and sex Riklie and Jones, 1991; 2013) Flexibility within the recommended range for age and sex Riklie and Jones, 1991; 2013)	0.0 0.5
	Balance	One-Leg Stand Test	≤20 s (Bohannon, 1994) ≥21 s (Bohannon, 1994)	0.0 0.5
Psychological	Depressive symptoms	*Geriatric Depression Scale* (GDS-15)	11 to 15 points (Yesavage et al., 1982) 6 a 10 points (Yesavage et al., 1982) 0 to 5 points (Yesavage et al., 1982)	0.0 1.0 2.0
Cognition	Global cognitibe screening	MoCA test	Score below the cutoff according to education level (Nasreddine et al., 2005) Score above the cutoff according to education level (Nasreddine et al., 2005)	0.0 2.0
Total				0 to 10 points

Physical variables assessed included: weight (kg), height (cm), body mass index (BMI, kg/m^2^), current body fat percentage (%), muscle mass (%), visceral fat (%), basal metabolic rate (kcal), body age (years), handgrip strength (kgf), lower limb strength (number of repetitions), functional mobility (seconds), abdominal circumference (cm), waist circumference (cm), thigh circumference (cm), calf circumference (cm), systolic and diastolic blood pressure (mmHg), heart rate (bpm), flexibility (cm), and balance (seconds). Details of this evaluation can be found at: https://doi.org/10.7910/DVN/UI5SGL.

### 2.2 Intrinsic capacity calculation

The selection of indicators for each domain to calculate intrinsic capacity was guided by the following criteria: predictors of functional decline and health during aging; preferably continuous scoring; ability to detect both low and high capacities in one of the five domains; ease of administration and incorporation into clinical and research practice. Subsequently, a group discussion among researchers was conducted to prioritize the most relevant available variables. These criteria were established because there is not yet a widely established standard for the use of variables in the calculation of the intrinsic capacity score.

The following assessments were used for each domain of intrinsic capacity.

1. **Vitality domain** (handgrip strength, BMI, and self-reported questions);
2. **Sensory domain** (self-reported questions extracted from ICOPE);
3. **Locomotor domain** (Timed Up and Go Test, Sit-to-Stand Test, Sitting and Reaching Test, and one-legged balance test);
4. **Psychological domain** (GDS-15);
5. **Cognitive domain** (Montreal Cognitive Assessment)

In the locomotion domain, for the Timed Up and Go test (Podsiadlo and Richardson, 1991), a value of 0.5 points was assigned for times under 10 s, and a value of 0 was assigned for times over 10 s. For the sit-to-stand test (Rikli and Jones, 1991; Rikli and Jones, 2013), a value of 0 was assigned for the number of repetitions below the age- and sex-specific threshold, and a value of 0.5 was assigned for the number of repetitions equal to or above the threshold. In the sit-and-reach test, a score of 0.5 was given for flexibility within the recommended range for age and sex, and a score of 0 for flexibility outside the recommended range. Finally, in the one-leg balance test, values of 20 s or less received a score of 0, and values of 21 s or more received a score of 0.5. The locomotion score was obtained by summing the values from the Timed Up and Go test, sit-to-stand test, sit-and-reach test, and one-leg balance test, with the final score ranging from 0 to 2.

In the vitality domain, for BMI, values below 22 kg/m^2^ and above 27 kg/m^2^ received a score of 0, while values between 22 and 27 kg/m^2^ received a score of 0.5 (Lipschitz, 1994). For handgrip strength (HGS), a score of 0 was assigned for values below 27 kgf for men and below 16 kgf for women. A score of 0.5 was assigned for values equal to or above 27 kgf for men and 16 kgf for women (Cruz-Jentoft et al., 2019). Weight loss was scored as follows: 0 for weight loss of 3 kg or more in the past 3 months and 0.5 for weight loss of less than 3 kg or no weight loss. For the variable appetite loss, a score of 0 was assigned for “yes” responses and 0.5 for “no” responses. The vitality score was obtained by summing the values for BMI, HGS, weight loss, and appetite loss, with the final score ranging from 0 to 2.

In the cognition domain, following the administration of the MoCA test (Nasreddine et al., 2005; Freitas et al., 2013), a score of 0 was assigned for results below the cut-off point according to educational level, and a score of 2 for results above the cut-off point. Thus, the final score for the cognition domain ranged from 0 to 2.

For the psychological domain, based on the GDS-15 scale (Yesavage et al., 1982), a score of 0 was assigned for values between 11 and 15; an intermediate score of 1 for values between 6 and 10; and the maximum score of 2 points for values between 0 and 5. Therefore, the final score for the psychological domain ranged from 0 to 2.

In the sensory domain, self-reported questions were used to establish scores for vision and hearing (WHO, 2017). For vision, a score of 0 was assigned to those who have difficulty seeing far or near and wear prescription glasses; the highest score of 1 was assigned to those who have no difficulty seeing far or near and do not wear prescription glasses. For hearing, a score of 0 was assigned to those who have difficulty hearing whispers and do not use hearing aids; the highest score of 1 was assigned to those who have no difficulty hearing whispers and do not use hearing aids.

The final score for the sensory domain was obtained by summing the scores for vision and hearing, with the total score ranging from 0 to 2.

The composite intrinsic capacity score will be calculated by summing the scores of each domain, totaling 10 points.

CI = locomotor (2 points) + vitality (2 points) + cognition (2 points) + psychological (2 points) + sensorial (2 points).

Total = 10 points.

The calculation of Intrinsic Capacity (IC) was based on previous studies, summing the scores of the individual domains without weighting, on a scale ranging from 0 to 2 points (Tay et al., 2023; Gutiérrez-Robledo et al., 2019; Ma et al., 2021; Muneera et al., 2022; Tay et al., 2022; Nagae et al., 2023; Muneera et al., 2023). Based on the composite intrinsic capacity calculation, the following classification categories were defined: 0–4 points as low intrinsic capacity, 5–8 points as moderate intrinsic capacity, and 9–10 points as high intrinsic capacity (López-Ortiz et al., 2022).

### 2.3 Intervention

After the pre-intervention data collection, the participants began the multicomponent training program. This 12-week program included both individual (strength training and aerobic exercises) and group sessions (10–20 participants), lasting 45 min each, five times per week. Exercise prescriptions were based on the guidelines of the American College of Sports Medicine (Nelson et al., 2007) and the Brazilian Ministry of Health (Brasil, 2021). The program offered the following modalities: strength training, aerobic exercises, circular dance, Chinese therapeutic gymnastics, Pilates matwork, gerontomotricity, hiking, maintenance gymnastics, shuttlecock sports, aquatic activities, and Nordic walking.

The selection of these modalities was conducted by a qualified training team in a personalized manner, respecting individual needs based on a prior physical assessment. Strength training and cardiovascular training were recommended for at least two sessions per week, while other activities could be performed up to three times per week. During the intervention period, the training team remained the same, and for most modalities, the sessions were led by a specific trainer. Additionally, participant preferences were taken into account to maximize engagement in the program.

Adherence monitoring was conducted using a specific diary in which participants recorded the date of each training session. After the 13th session, exercise intensity and volume parameters were adjusted, with progression defined by a 5% increase in total load accompanied by a reduction in the number of repetitions, from 15 to 10. These adjustments were individualized based on participants’ responses, considering their tolerance to effort and performance during previous sessions.

During cardiovascular training sessions, pre- and post-activity blood pressure and participant heart rate were monitored to enable exercise prescription and intensity adjustment based on individualized target zones (40%–50% of HR_max_ for maintenance, 60%–70% for fat mass reduction, and 75%–85% for general physical conditioning), tailored to each older adult’s functional and clinical profile. Additionally, parameters including speed, perceived exertion, exercise duration, incline (when applicable), and estimated calories were recorded across various aerobic equipment (cycle ergometer, treadmill, elliptical, and rowing machine).

The detailed tracking of the sessions allowed for strict control of the regularity and engagement of participants with the program, contributing to adherence and efficient management of progression. The training schedule, which included activities planned for both morning and afternoon periods, was organized with pre-established times. Participants could choose the day and time of the week to attend sessions, according to the period in which they attended the program. The schedule with activities and times, as well as the complete details of the intervention, can be found in the Supplementary Material (https://doi.org/10.7910/DVN/UI5SGL).

During the program, participants also received health education through lectures by specialists. Topics included chronic disease management, healthy eating, reducing sedentary behavior, medication safety, and other relevant themes.

### 2.4 Intervention follow-up

During the 12 weeks following the intervention, all older adults were reassessed and questioned about the modalities they practiced, as well as the frequency and duration of their sessions on each day attended.

### 2.5 Statistical analysis

Data analysis was performed using SPSS version 28.0, and an intention-to-treat approach was used, including all participants initially enrolled, regardless of adherence to the intervention. Quantitative variables were described using means and standard deviations or medians and interquartile ranges. Categorical variables were described using absolute and relative frequencies. The comparison between pre- and post-intervention moments was evaluated using the Generalized Estimating Equations (GEE) model. A linear model was applied for variables with a normal distribution, a logarithmic transformation model for variables with asymmetric distributions, an ordinal logistic model for ordinal variables, and a binary logistic model for dichotomous variables. A significance level of 5% (p < 0.05) was adopted. The raw data are available at: https://doi.org/10.5281/zenodo.14002534.

The standardized effect size (SES) was used to assess the magnitude of the intervention effect. According to Cohen (1988), a value below 0.5 indicates a small effect, between 0.5 and 0.8 a moderate effect, and above 0.8 a large effect (COHEN, 1988).

## 3 Results

### 3.1 Main results

The final sample consisted of 43 older adults, with a mean age of 67.7 years (standard deviation ± 4.3). The majority were women (86.0%), married or living with a partner (44.2%), living alone (39.5%), and had completed high school or higher education (23.3%). Regarding family and social arrangements, the mean number of children was 2.0 (standard deviation ± 1.2), and most participants reported having a support network (93.0%); however, only 20.9% mentioned attending social spaces for older adults.

Regarding the multicomponent training, more than half of the older adults attended the program three times per week (55.0%) and performed 90 min of physical activity per day attended (62.5%). Among the exercise modalities, the most practiced were Chinese therapeutic gymnastics (87.5%), followed by gerontomotricity (80.0%) and maintenance gymnastics (70.0%). Among the participants, 93% adhered to the program and completed the pre- and post-assessments, and only three participants did not complete the final assessment due to leaving the program.

When comparing the pre- and post-intervention moments in the composite intrinsic capacity classification and specific domains, a reduction in the proportion of participants classified as having “low” capacity was observed, decreasing from 7.0% initially to 0.0% after the intervention (p = 0.018). Conversely, the “high” capacity classification increased from 4.7% to 20.0%, indicating a significant improvement in this variable among participants (Table 2).

**TABLE 2:** Comparison between baseline and follow-up in the composite intrinsic capacity score and domains.

Variables	Baseline	Follow-up	p-value	Effects-size (95% CI)
	n (%)/medians (P25 – P75)	n (%)/medians (P25 – P75)		
CIC Classification			0.018	0.38 (0.06–0.69)
Low	3 (7.0)	0 (0.0)		
Moderate	38 (88.4)	32 (80.0)		
High	2 (4.7)	8 (20.0)		
CIC Domains				
Sensory (DS)	1 (0–1)	1 (0–1)	0.993	0.00 (−0.31 to 0.31)
Psychological (DP)	2 (2–2)	2 (2–2)	0.100	0.23 (−0.09 to 0.54)
Cognitive (DC)	2 (0–2)	2 (2–2)	<0.001	0.61 (0.27 to –0.94)
Vitality (DV)	1.5 (1.5–2)	1.5 (1.5–2)	0.045	0.31 (0.01 to 0.63)
Locomotion (DL)	1.5 (1.0–1.5)	2.0 (1.5–2.0)	<0.001	1.12 (0.72 to 1.52)

Note: Values are presented as medians with interquartile ranges (P25–P75).

Abbreviations: DS, sensory domain; DP, psychological domain; DC, cognitive domain; DV, vitality domain; DL, locomotion domain; CIC, composite intrinsic capacity.

Regarding the intrinsic capacity domains, the median in the cognitive domain (DC) showed a significant increase, from a median of 0–2 to 2–2 (p < 0.001). The vitality domain (DV) also exhibited a statistically significant change (p = 0.045), as did the locomotion domain (DL), which increased from 1.0 to 1.5 to 1.5–2.0 (p < 0.001). However, no significant changes were observed in the sensory domain (SD) and psychological domain (DP).

### 3.2 Secondary results

Comparisons between the pre- and post-intervention periods regarding participants’ quality of life and self-perceived health are presented in Table 3. For self-perceived health, the proportion of participants rating their health as “poor” decreased from 7.0% pre-intervention to 0.0% post-intervention (p = 0.002). The proportion of those perceiving their health as “fair” decreased slightly, from 18.6% to 15.0%, while the “good” category showed a more significant reduction, from 65.1% to 50.0%. Notably, the proportion of participants rating their health as “excellent” increased from 9.3% to 35.0% after the intervention, indicating an improved perception of health among participants.

**TABLE 3:** Comparison between baseline and follow-up in quality of life and self-perceived health.

Variables	Baseline	Follow-up	p-value
	n (%)/mean	n (%)	
Self-perceived health			0.002
Poor	3 (7.0)	0 (0.0)	
Fair	8 (18.6)	6 (15.0)	
Good	28 (65.1)	20 (50.0)	
Excellente	4 (9.3)	14 (35.0)	
Quality of life			
SF6D	0.84 ± 0.08	0.88 ± 0.08	0.004

Note: Values are presented as frequencies and percentages (n; %) or as means and standard deviations (±).

Regarding quality of life, the mean SF-6D index score increased from 0.84 ± 0.08 to 0.88 ± 0.08, demonstrating a statistically significant improvement (p = 0.004). These results suggest that the intervention positively impacted subjective quality of life and participants’ self-perceived health.

In the analysis of secondary variables related to movement behavior, a significant increase in physical activity levels was observed (814.5 ± 475.2 min to 1,332 ± 798.3 min; p < 0.001), including a significant rise in the classification of “active” participants (46.5%–77.5%, p = 0.004). Sedentary behavior time decreased (245.5 ± 126.0 min to 225.7 ± 144.8 min), although this reduction was not statistically significant.

Regarding functionality, the CPF scale score increased from 22.0 ± 2.9 to 22.6 ± 3.1 points, but this change was not significant. However, the classification showed a significant improvement (p = 0.021), particularly in the “elevated” category.

In terms of clinical variables, a significant reduction in body fat percentage was identified (37.5 ± 9.5 to 35.5 ± 9.2, p < 0.001), along with a significant improvement in its classification (p = 0.015). Additionally, muscle mass showed a significant increase (26.6 ± 4.5 to 27.8 ± 4.5, p < 0.001).

For anthropometric measures, significant reductions were observed in waist circumference (85.2 ± 10.7 cm to 83.9 ± 10.1 cm, p < 0.001) and abdominal circumference (93.1 ± 10.9 cm to 90.7 ± 10.5 cm, p = 0.001). Similarly, decreases were noted in systolic blood pressure (126.6 ± 15.0 mmHg to 125.1 ± 12.9 mmHg), diastolic blood pressure (79.1 ± 8.9 mmHg to 77.7 ± 8.7 mmHg), and heart rate (73.2 ± 12.4 bpm to 70.7 ± 11.6 bpm).

Finally, a significant improvement was observed in the overall score of the quality-of-life scale (0.84 ± 0.08 to 0.88 ± 0.08, p = 0.004) (Table 4).

**TABLE 4:** Comparison between baseline and follow-up um functional and clinical variables.

Variables	Baseline	Follow-up	p
	Medians (P25 – P75) [mean]	Medians (P25 – P75) [mean]	
IPAQ Total	690 (505–1,080) [814,5]	1,225 (832.5–1,397) [1332.6]	<0.001
IPAQ Classification – n (%)			0.004
Insufficiently active	23 (53.5)	9 (22.5)	
Active	20 (46.5)	31 (77.5)	
Sedentary behavior (min)	225 (150–360) [245,5]	187.5 (120–263.1) [225,7]	0.460
Sedentary Classification – n (%)			0.498
Low	41 (95.3)	37 (92.5)	
High	2 (4.7)	3 (7.5)	
CPF Score	22.0 ± 2.9	22.6 ± 3.1	0.230
CPF classification – n (%)			0.021
Low	3 (7.0)	3 (7.5)	
Moderate	22 (51.2)	12 (30.0)	
High	18 (41.9)	25 (62.5)	
Body fat (%)	37.5 ± 9.5	35.5 ± 9.2	<0.001
Muscle mass (%)	26.6 ± 4.5	27.8 ± 4.5	<0.001
Abdominal circumference (cm)	93.1 ± 10.9	90.7 ± 10.5	0.001
Righ calf circumference (cm)	36.0 ± 3.2	35.7 ± 3.1	0.219
Left calf circumference (cm)	35.9 ± 3.1	35.7 ± 3.2	0.435
Calf Classification – n (%)			0.879
Normal	40 (93.0)	37 (92.5)	
Reduced	3 (7.0)	3 (7.5)	
BP Classification – n (%)			0.524
Excellent	13 (30.2)	15 (37.5)	
Normal	13 (30.2)	9 (22.5)	
Prehypertension	8 (18.6)	9 (22.5)	
Stage I Hypertension	9 (20.9)	7 (17.5)	
Heart rate (bpm)	73.2 ± 12.4	70.7 ± 11.6	0.100

Note: Values are presented as medians with interquartile ranges (P25–P75), means and standard deviations (±), or frequencies and percentages (n; %).

Abbreviations: IPAQ, International Physical Activity Questionnaire; CPF, Composite Physical Function; BP, Blood Pressure; SF6D - Short Form 6 Dimensions.

## 4 Discussion

This study investigated changes in intrinsic capacity and functional and psychosocial aspects among older adults participating in a multicomponent physical exercise program. Over the analyzed period, the program significantly improved the classification of intrinsic capacity (IC) and its domains. Additionally, it positively influenced functional and psychosocial variables.

Regarding intrinsic capacity measurement, a higher percentage of older adults were classified in the high IC category after the intervention, and no participants fell into the low IC category. Literature indicates that higher IC scores are associated with a reduced risk of frailty among robust older adults, regardless of age, comorbidities, and social vulnerability, as well as a lower likelihood of health deterioration, falls, and functional decline (Tay et al., 2023; Yu et al., 2023).

Significant improvements were observed in the cognition, vitality, and locomotion domains. Previous studies suggest multicomponent exercise positively affects global cognition (Stern et al., 2019; Bisbe et al., 2020), particularly when aerobic exercise is part of the intervention (Venegas-Sanabria et al., 2022). This effect is attributed to the release of cytokines, such as ciliary neurotrophic factor (CNTF), leukemia inhibitory factor (LIF), and vascular endothelial growth factor (VEGF), which are related to neuronal plasticity (Duzel et al., 2016; Berg and Bang, 2005).

In the vitality domain, participants experienced a reduction in BMI, although no significant changes were observed in handgrip strength. Multicomponent exercise programs may yield improvements in this variable when stimuli are sustained over time. The 12-week duration of our study may have limited the detection of significant changes in this regard (Rodrigues et al., 2022; Monteiro et al., 2019; Nogueira et al., 2017).

Significant enhancements were also noted in mobility, lower limb strength, flexibility, and balance, which comprise the locomotor domain. Strength training stimulates muscle fibers, resulting in increased muscle strength (Monteiro et al., 2021; Marques et al., 2023). Simultaneously, improved muscle fiber elasticity promotes flexibility and range of motion (Rodrigues et al., 2022). The exercises incorporated daily life movements, such as standing up, walking, and agility drills, which dynamically challenge the body and likely contributed to improvements in mobility and balance (Rodrigues et al., 2023). Our findings align with previous research demonstrating the benefits of multicomponent exercise on locomotor domains (Coelho-Junior et al., 2017; Tarazona-Santabalbina et al., 2016; Freiberger et al., 2012; Kang et al., 2015; Toto et al., 2012).

The locomotor domain showed a large effect size (es = 1.12), indicating a meaningful change following the intervention. Although well-established reference values for clinically significant changes in the domains of Intrinsic Capacity are still lacking, this result suggests a relevant improvement, consistent with findings from similar multicomponent exercise programs previously reported in the literature (Sanchez-Sanchez et al., 2024).

Physical activity levels also increased significantly, with many participants classified as highly active post-intervention. Accumulating sufficient physical activity is associated with health benefits across all ages (Warburton et al., 2006; Nelson et al., 2007; Sui et al., 2007; Vogel et al., 2009).

Body composition results, particularly in abdominal circumference, mirror findings from similar Brazilian studies (Oliveira et al., 2021). In Korea, combined training interventions also showed positive effects on body mass, fat percentage, and BMI among older women (Lee et al., 2015). A recent systematic review recommends 12-week combined exercise prescriptions, 3–4 days per week, to achieve body composition changes in older adults, aligning with our intervention design (Wu et al., 2023).

The program also demonstrated significant benefits in subjective variables, especially quality of life and self-rated health. These improvements can be attributed to enhanced independence, as participants found daily tasks easier to perform with less effort and assistance. Consequently, older adults perceive their health and quality of life more positively (Roberts and Adams, 2017; Whitehurst et al., 2005; Maung et al., 2022; Atad and Caspi, 2020).

Additionally, the program fostered social interaction, allowing participants to express emotions, thoughts, and ideas, strengthening their social and cultural bonds (Pucci et al., 2012). This aligns with the WHO’s Decade of Healthy Aging framework, emphasizing meaningful societal participation in later life (Who, 2020).

A notable limitation of this study is its non-randomized design and convenience sampling, which may introduce selection bias and limit the generalizability of the results. However, it is important to highlight that this study was specifically designed to evaluate older adults already participating in a community-based physical activity program, representing a specific subgroup within the older population. Therefore, the sample was intentionally chosen to reflect this group, aiming to generate evidence applicable to strategies for maintaining and optimizing intrinsic capacity in this context. This sample characteristic reflects the pragmatic focus of the study, centered on evaluating interventions in real-world implementation settings, such as community health programs, where voluntary adherence is an expected condition. Thus, although generalization to the entire older adult population should be made with caution, the findings are highly relevant for public policies and existing community programs.

While the tailored approach to exercise prescription allows for individualized interventions, it also introduces variability in implementation and adherence, which may impact outcomes. Future studies should address this limitation by employing randomized controlled trial designs with larger and more diverse samples to enhance the robustness and generalizability of the findings. Additionally, long-term follow-up could clarify sustained benefits and explore additional mechanisms underlying the observed improvements. Integration with emerging technologies, such as wearable devices and AI-based monitoring systems, may further optimize exercise prescriptions and adherence, ultimately contributing to better health outcomes in the aging population. Another limitation is the short duration of the intervention (12 weeks), which may not fully capture the long-term effects of the multicomponent exercise program on intrinsic capacity. Extending the duration of the intervention and incorporating follow-up assessments could provide a more comprehensive understanding of the sustained benefits and potential cumulative effects of the exercise program.

An additional limitation concerns the lack of a standardized and validated scoring system for intrinsic capacity assessment. Currently, there is no consensus regarding scoring criteria or specific weighting for individual domains. This methodological gap reflects the still-emerging nature of the field, where different measurement approaches are being tested and compared. Our selection of the simple summation method was based on its widespread adoption in current literature and the need to maintain comparability with previous studies. We acknowledge that future psychometric validations may lead to the development of more refined scoring systems that are sensitive to population-specific variations in intrinsic capacity.

## 5 Conclusion

The multicomponent exercise program demonstrated significant improvements in the intrinsic capacity of community-dwelling older adults over a 12-week period. Notable advancements were observed in cognitive function, vitality, and locomotion, underscoring the program’s efficacy in promoting healthy aging. Beyond physical factors such as increased physical activity levels, enhanced functionality, and improved body composition, participants also reported subjective benefits in quality of life and self-perceived health. These findings highlight the critical role of tailored and supervised exercise interventions in enhancing both physical and psychosocial dimensions of health, contributing to healthier aging trajectories. Future research should explore the long-term benefits and underlying mechanisms driving these improvements to further refine and optimize such interventions.

## Data Availability

The datasets presented in this study can be found in online repositories. The names of the repository/repositories and accession number(s) can be found in the article/Supplementary Material.
